# Characterisation of endogenous A_2A_ and A_2B_ receptor-mediated cyclic AMP responses in HEK 293 cells using the GloSensor™ biosensor: Evidence for an allosteric mechanism of action for the A_2B_-selective antagonist PSB 603

**DOI:** 10.1016/j.bcp.2017.10.013

**Published:** 2018-01

**Authors:** Joelle Goulding, Lauren T. May, Stephen J. Hill

**Affiliations:** a Division of Physiology, Pharmacology and Neuroscience, School of Life Sciences, University of Nottingham, Nottingham NG7 2UH, UK; b Centre of Membrane Proteins and Receptors (COMPARE), University of Birmingham and University of Nottingham, Midlands, UK

**Keywords:** A_2A_AR, A2A adenosine receptor, A_2B_AR, A2B adenosine receptor, BAY 60-6583, (2-[[6-Amino-3,5-dicyano-4-[4-(cyclopropylmethoxy)phenyl]-2-pyridinyl]thio]-acetamide, HEK293G, Human Embryonic Kidney 293 cell line stably expressing the GloSensor™ biosensor, DMEM, Dulbecco modified eagles medium, FCS, fetal calf serum, HBSS, HEPES buffered saline solution, NECA, 5′-(N-Ethylcarboxamido)adenosine, CGS 21680, 4-[2-[[6-Amino-9-(N-ethyl-β-d-ribofuranuronamidosyl)-9H-purin-2-yl]amino]ethyl]benzene propanoic acid hydrochloride, XAC, Xanthine amine congener, PSB 603, 8-[4-[4-(4-chlorophenzyl)piperazide-1-sulfonyl)phenyl]]-1-propylxanthine, ZM 241385, 4-(2-[7-Amino-2-(2-furyl)[1,2,4]triazolo[2,3-a][1,3,5]triazin-5-ylamino]ethyl)phenol, SCH 58261, 2-(2-Furanyl)-7-(2-phenylethyl)-7H-pyrazolo [4,3-e] [1,2,4]triazolo[1,5-c]pyrimidin-5-amine, cAMP, cyclic AMP, ADA, adenosine deaminase, Adenosine (PubChem CID: 60961), BAY 60-6583 (PubChem CID: 11717831), CGS 21680 (PubChem CID: 10256643), Forskolin (PubChem CID: 47936), NECA (PubChem CID: 448222), PSB 603 (PubChem CID: 44185871), SCH 58261 (PubChem CID: 176408), XAC (PubChem CID: 5697), ZM 241385 (PubChem CID: 176407), Adenosine receptor, Cyclic AMP, Kinetics, Allosterism, PSB 603, A_2B_ receptor

## Abstract

Endogenous adenosine A_2B_ receptors (A_2B_AR) mediate cAMP accumulation in HEK 293 cells. Here we have used a biosensor to investigate the mechanism of action of the A_2B_AR antagonist PSB 603 in HEK 293 cells. The A_2A_ agonist CGS 21680 elicited a small response in these cells (*circa* 20% of that obtained with NECA), suggesting that they also contain a small population of A_2A_ receptors. The responses to NECA and adenosine were antagonised by PSB 603, but not by the selective A_2A_AR antagonist SCH 58261. In contrast, CGS 21680 responses were not antagonised by high concentrations of PSB 603, but were sensitive to inhibition by SCH 58261. Analysis of the effect of increasing concentrations of PSB 603 on the response to NECA indicated a non-competitive mode of action yielding a marked reduction in the NECA E_MAX_ with no significant effect on EC_50_ values. Kinetics analysis of the effect of PSB 603 on the A_2B_AR-mediated NECA responses confirmed a saturable effect that was consistent with an allosteric mode of antagonism. The possibility that PSB 603 acts as a negative allosteric modulator of A_2B_AR suggests new approaches to the development of therapeutic agents to treat conditions where adenosine levels are high.

## Graphical abstract

**Figure:**
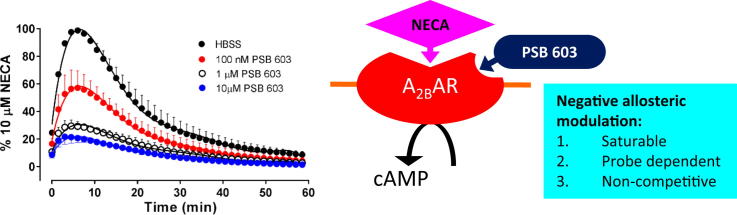

## 1 Introduction

Adenosine acts via four (A_1_, A_2A_, A_2B_ and A_3_) specific G protein-coupled receptors (GPCRs) [1]. The A_1_ and A_3_ receptors couple to Gi/o proteins and inhibit adenylyl cyclase activity whilst the A_2A_ and A_2B_ receptors preferentially couple to Gs proteins and stimulate the formation of cyclic AMP (cAMP) [1], [2], [3]. The crystal structure of the A_2A_ receptor (A_2A_AR) in both antagonist [4] and agonist [5] bound conformations has been determined in recent years. The adenosine A_2B_ receptor (A_2B_AR), which is closely related to the A_2A_AR, is the least well defined of the four adenosine receptors and has low affinity for the endogenous agonist, adenosine [3], [6]. A_2B_ARs have been reported to have important roles in inflammation, fibrosis, angiogenesis and tumour progression [3], [7], [8], [9], [10], [11] making them an important therapeutic target for drug discovery.

Whilst there are a number of selective ligands available for the A_1_, A_3_ and A_2A_ receptors, these are more limited for A_2B_AR [3], [12], [13], [14], [15]. However, a selective A_2B_AR antagonist has been recently developed (PSB 603) which has also been used as a radioligand (^3^H-PSB-603) [16]. This compound has been used to investigate the amino acids involved in the interaction of agonists and antagonists with A_2B_AR [6]. This study showed that whilst Trp247, Val250 (both in transmembrane 6;TM6), and Ser279 (TM7) were important for the binding of nucleoside-based agonists, Leu81 (TM3), Asn186 (TM5) and Val250 (TM6) were crucial for binding of the xanthine-derived antagonist PSB 603 [6]. These data suggest that PSB 603 may bind to a different set of amino acids to those used by the endogenous ligand adenosine, and this raises the possibility of an allosteric mechanism of action.

Allosteric ligands bind to a topographically distinct site (allosteric) from that occupied by the endogenous agonist (orthosteric site) and elicit a conformational change that can lead to alterations in the affinity or efficacy of the ligand occupying the orthosteric binding site [17], [18], [19], [20]. Key features of an allosteric mechanism of action are that the effect is saturable (i.e. reaches a limiting maximal effect), can depend on the specific ligand occupying the orthosteric site (probe dependence) and provides scope for both positive and negative effects on ligand binding and/or function [17], [18], [19], [20]. An allosteric mechanism of action can provide a drug with a number of potential advantages such as introducing greater selectivity for the target, producing an effect that may depend on concurrent binding of the natural ligand and, in the case of negative allosteric regulators, a non-competitive effect that is resistant to high concentrations of the endogenous orthosteric agonist. This may have advantage for A_2B_AR directed therapeutics that are designed to address conditions such as ischemia and inflammation where levels of adenosine may be very high.

In the present study we have characterised the cAMP responses elicited by endogenous A_2B_AR expressed in HEK 293 cells [21] with particular reference to the potential for allosteric interactions. We also provide evidence for a minor population of endogenous A_2A_AR in this cell line. To help with this characterisation, we have used the following adenosine receptor antagonists (their respective Ki values for A_2A_AR and A_2B_AR given in parentheses): XAC (1 nM [22], 73 nM [23]); ZM 241385 (1.4 nM, 32 nM [23]); SCH 58261 (0.6 nM, 5011 nM [24]) and PSB 603 (Ki > 10,000 nM, 0.5 nM [16]). We have studied real-time kinetic changes in cAMP levels using the GloSensor™ biosensor (Promega) in intact living cells [25], [26]. The GloSensor™ technology is based on an engineered form of firefly luciferase encompassing a cAMP-binding domain from protein kinase A (RIIβB; [26]). Upon binding of cAMP, in the presence of the GloSensor™ substrate [26], the resultant conformational change in the GloSensor™ biosensor leads to light emission that can be detected by an automated plate-reader. This assay lends itself nicely to the study of GPCR mediated cAMP modulation in both endogenous and over expressed systems. For example, it has been used to study the Gα_s_-coupled β2-adrenergic receptor found endogenously in HEK293 cells [27] or over-expressed in HEK293 cells, and used to dissect intracellular signalling [28]. Furthermore, Gα_i/o_-coupled responses can be determined from their ability to inhibit forskolin-stimulated cAMP responses (e.g. for the metabotropic glutamate receptor expressed in CHO K1 cells [29]) or reduce basal levels of cAMP (e.g. the succinate receptor 1 in HEK293 cells [30]). Here we have used the GloSensor™ biosensor to study the pharmacological profile, and mechanism of action of PSB 603 as an antagonist, of G_s_-coupled A_2B_ARs endogenously expressed in HEK293 cells.

## 2 Materials and methods

### 2.1 Cultured cells

The cAMP GloSensor™ (20F) biosensor [26] expressed in HEK293 (HEK293G) cells was obtained from Promega (Madison, WI). HEK293G cells were maintained in Dulbecco modified eagles medium (DMEM) supplemented with 2 mM L-glutamine, 10% FCS (fetal calf serum) and 200 μg/ml hygromycin B at 37 °C 5% CO_2_. Once confluent, cells were dislodged from the flask surface by gentle shaking after incubation in 0.25% trypsin and cell pellet formed following 5 min 1000 g centrifugation. For the GloSensor™ assay, cells were re-suspended in DMEM supplemented with 2 mM L-glutamine and 10% FCS and seeded at a density of 35000 cells/well on poly-l-lysine treated clear bottomed white walled 96 well plates. Cells were incubated at 37°C 5% CO_2_ overnight prior to assay.

### 2.2 GloSensor™ assay

The GloSensor™ assay was carried out as per manufacturer’s instructions (Promega, Madison, WI, USA). Briefly, this was as follows; Media was aspirated and cells were incubated in 100 µl HBSS (HEPES buffered saline solution pH 7.45; Sodium pyruvate 2 mM, NaCl 145 mM, d-Glucose 10 mM, KCL 5 mM, MgSO_4_·7H_2_O 1 mM, HEPES 10 mM, CaCl_2_ 1.7 mM, NaHCO_3_ 1.5 mM) containing 4–6% GloSensor™ cAMP reagent and incubated for 2 h at final experimental temperature of 35°C. Luminescence was measured on an EnVision® Multilabel Plate Reader (Perkin Elmer, Massachusetts, USA) continuously over 60 min, averaging 1 read per well every 1.5 min, following the addition of 100 µl HBSS in the presence or absence of Forskolin (10 nM–10 µM), NECA (5′-(N-Ethylcarboxamido)adenosine, 10 nM-30 µM), Adenosine (100 nM-100 µM), BAY 60-6583 (2-[[6-Amino-3,5-dicyano-4-[4-(cyclopropylmethoxy)phenyl]-2-pyridinyl]thio]-acetamide, 1 pM-30 μM) or CGS 21680 (4-[2-[[6-Amino-9-(N-ethyl-β-D-ribofuranuronamidosyl)-9H-purin-2-yl]amino]ethyl]benzene propanoic acid hydrochloride, 30 nM-30 µM). Antagonist action was monitored following 30 min pre-incubation with HBSS in the presence or absence of XAC (xanthine amine congener), PSB 603 (8-[4-[4-(4-Chlorophenzyl)piperazide-1-sulfonyl)phenyl]]-1-propylxanthine), ZM 241385 (4-(2-[7-Amino-2-(2-furyl)[1,2,4]triazolo[2,3-a][1,3,5]triazin-5-ylamino]ethyl)phenol or SCH 58261 (2-(2-Furanyl)-7-(2-phenylethyl)-7H-pyrazolo [4,3-e][1,2,4] triazolo[1,5-c]pyrimidin-5-amine).

### 2.3 Data analysis

Determinations of agonist potency, antagonist affinity and equilibrium dissociation constants were made by fitting data within GraphPad Prism version 5.03 for Windows (GraphPad Software, San Diego California USA, www.graphpad.com).

To obtain the antagonist equilibrium dissociation constants (*K*_B_) a modified form of the Gaddum equation was used as described by Lazareno and Birdsall [31]:

$$K_{\text{B}}=\frac{{\text{IC}}_{50}}{[\text{A}]/{\text{EC}}_{\text{F}}-1}$$

where IC_50_ is the molar concentration of antagonist (B) required to decrease by 50% the response mediated by the fixed molar concentration of agonist (A) in the absence of antagonist; and EC_F_ the molar concentration of agonist that, in the absence of antagonist, mediated the same response as that obtained in the presence of an IC_50_ concentration of antagonist. Agonist concentration response curves were simultaneously obtained (in the absence of antagonist). Estimated affinity values (K_B_) were also calculated from the shift of the agonist concentration response curves in the presence of a fixed concentration of antagonist using the following equation:

$$\mathrm{DR}=1+\frac{\left[B\right]}{K_{B}}$$

where DR (dose ratio) is the ratio of the agonist concentration required to stimulate an identical response in the presence and absence of antagonist, [B].

Statistical significance was determined, where appropriate, using Student’s *t*-test, linear regression or one-way ANOVA with either Bonferroni’s (Table 1) or Dunnett’s (Table 2) multiple comparison test if p < .05 (statistically significant). A minimum of three independent experiments was undertaken for all experimental work. This was based on the variance of the data obtained and power analysis that predicted a high probability of observing small differences in measured parameters. For example, the probability of detecting a change in pEC_50_ of 0.5 (3-fold) with n = 3 is 0.89. For n = 4 this increased to 0.96.

**Table 1:** pIC_50_ and apparent dissociation equilibrium constants for adenosine A2 receptor antagonists.

Agonist		XAC	PSB 603	ZM 241385	SCH 58261
NECA	pIC_50_ IC_50_ (nM)	6.93 ± 0.10 (6) 134.8 ± 34.3	7.53 ± 0.22 (6) 49.8 ± 19.1	7.11 ± 0.10 (5) 84.4 ± 15.8	nd
	-Log K_B_ K_B_ (nM)	7.90 ± 0.10 (6) 14.4 ± 3.7	8.50 ± 0.22 (6) 5.3 ± 2.0	8.08 ± 0.10 (5) 9.0 ± 1.7	nd
CGS 21680	pIC_50_ IC_50_ (nM)	7.02 ± 0.09 (5) 103.9 ± 18.7	nd	8.85 ± 0.18 (7) 2.3 ± 0.9	7.64 ± 0.11 (8) 28.4 ± 5.8
	-Log K_B_ K_B_ (nM)	7.89 ± 0.09 (5) 14.0 ± 2.5	nd	9.72 ± 0.18^1^ (7) 0.3 ± 0.1	8.51 ± 0.11 (8) 3.8 ± 0.8
Adenosine	pIC_50_ IC_50_ (nM)	7.15 ± 0.12 (4) 97.0 ± 47.0	7.56 ± 0.43 (5) 140.9 ± 118.4	7.66 ± 0.14 (5) 28.1 ± 10.6	nd
	-Log K_B_ K_B_ (nM)	7.48 ± 0.12 (4) 45.6 ± 22.1	7.89 ± 0.43 (5) 66.3 ± 55.7	7.99 ± 0.14 (5) 13.2 ± 5.0	nd
BAY 60-6583	pIC_50_ IC_50_ (nM)	6.81 ± 0.10 (6) 173.8 ± 33.9	7.86 ± 0.13 (6) 17.4 ± 5.4	7.05 ± 0.16 (6) 119.8 ± 38.5	nd
	-Log K_B_ K_B_ (nM)	7.50 ± 0.10 (6) 35.6 ± 6.9	8.55 ± 0.13 (6) 3.6 ± 1.1	7.74 ± 0.16 (6) 24.5 ± 7.9	nd

IC_50_ values and apparent dissociation equilibrium constants (K_B_) were calculated from NECA, CGS 21680, adenosine and BAY 60-6583 competition curves alongside agonist concentration curves in the absence of antagonist. Data are expressed as mean ± S.E.M. of *n* separate experiments (shown in parentheses). ^1^The apparent equilibrium dissociation constant for ZM 241385 following CGS 21680 stimulation is significantly different to that observed following NECA, BAY 60-6583 and adenosine stimulation (p < .0001; 1-way ANOVA). nd – Not determined because of lack of antagonism (see Fig. 6).

**Table 2:** Agonist pEC_50_ and E_max_ values obtained in the presence of increasing concentrations of PSB 603.

	NECA		Adenosine		BAY 60-6583	
PSB 603 (nM)	pEC_50_	E_max_	pEC_50_	E_max_	pEC_50_	E_max_
0	5.77 ± 0.08	112.43 ± 3.08	4.97 ± 0.04	107.33 ± 3.67	6.39 ± 0.04	104.40 ± 5.14
30	5.66 ± 0.20	93.60 ± 8.38	4.56 ± 0.08^**^	99.97 ± 10.83	5.80 ± 0.18	82.23 ± 1.64
100	5.86 ± 0.13	53.59 ± 8.68^**^	4.34 ± 0.08^**^	67.72 ± 5.94^**^	5.34 ± 0.22^**^	68.66 ± 6.78
300	5.68 ± 0.25	43.22 ± 7.60^**^	4.03 ± 0.13^**^	57.36 ± 6.13^**^	4.44 ± 0.13^****^	99.35 ± 26.61

pEC_50_ and E_max_ values obtained for NECA and adenosine obtained in the presence of increasing concentrations of PSB 603. EMAX values are expressed as a percentage of the response obtained with 10 μM NECA, 100 μM adenosine or 10 µM BAY 60-6583. Significant differences to that seen in the absence of antagonist with each agonist are indicated (^**^p < .01, ^****^p < .0001, 1-way ANOVA). Data are expressed as mean ± S.E.M. of 4 (NECA), 7 (adenosine) or 3 (BAY 60-6583) separate experiments.

### 2.4 Drugs, chemical reagents and other materials

Glosensor™ cAMP Human Embryonic Kidney 293 cell line (Hek293G) and GloSensor™ cAMP Reagent were obtained from Promega (Wisconsin, USA). Forskolin, NECA, Adenosine, XAC and SCH 58261 were from Sigma-Aldrich (Missouri, USA). PSB 603, ZM 241385, BAY 60-6583 and CGS 21680 hydrochloride were from Tocris Bioscience (Bristol, UK). Adenosine Deaminase (ADA) was from Roche (Mannheim, Germany). Hygromycin B was from Invitrogen (Paisley, UK). L-glutamine, trypsin and FCS were from Lonza (Verviers, Belgium). All other chemicals were from Sigma-Aldrich (Missouri, USA).

## 3 Results

### 3.1 Kinetic profile of forskolin- and NECA- stimulated GloSensor™ luminescence in HEK293G cells

**Fig. 1:**
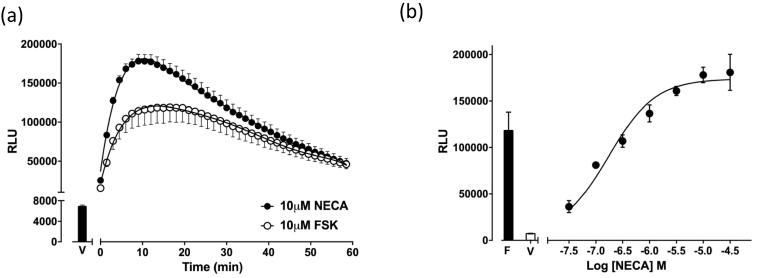
Forskolin – and NECA – stimulated cAMP Glosensor luminescence. (a) Glosensor luminescence time courses following stimulation by increasing forskolin (10 µM) or NECA (10 µM). Data are mean ± S.E.M. of triplicate determinations obtained in a single representative experiment and expressed as relative luminescence units (RLU). Similar data were obtained in 8 (Forskolin) and 21 (NECA) separate experiments. (b) Representative concentration response curve for NECA obtained from triplicate determinations in a single experiment. Bars show the peak luminescence response to 10 μM forskolin (F) and that seen with vehicle alone (V). Similar data were obtained in 21 separate experiments.

**Fig. 2:**
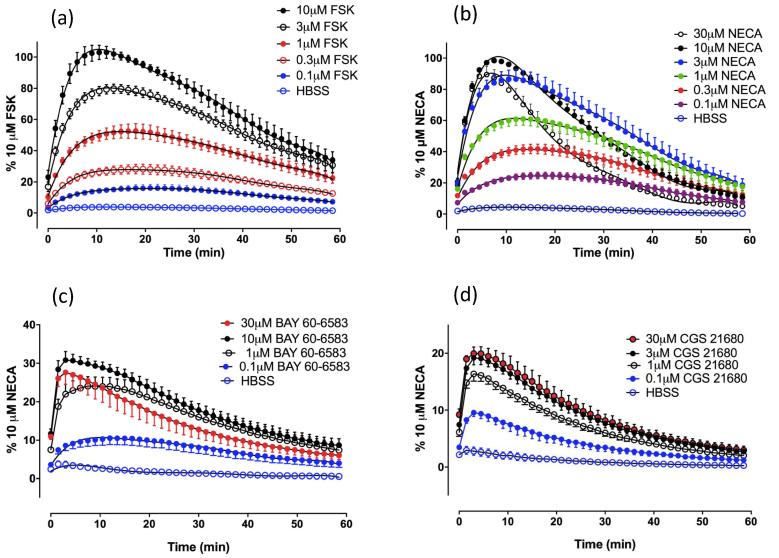
Kinetic profile of increasing concentrations of (a) forskolin, (b) NECA, (c) BAY 60-6583 or (d) CGS 21680 – stimulated cAMP Glosensor luminescence. Data are mean ± S.E.M. expressed as a percentage of the peak luminescence obtained in 8 (a), 21 (b), 3 (c) or 4 (d) separate experiments each performed in triplicate. (For interpretation to colours in this figure, the reader is referred to the web version of this paper.)

The effect of direct activation of adenylyl cyclase by forskolin on cAMP production and subsequent GloSensor™ luminescence in HEK293G cells is shown in Figs. 1a and 2a. Forskolin (10 µM) stimulated a concentration-dependent increase in luminescence which was followed (after achievement of the peak response) by a slow decline in the luminescence signal (Figs. 1a and 2a). We have previously reported that the HEK-293 cells express an endogenous adenosine A_2B_AR [21]. Stimulation of this receptor via the non-selective adenosine receptor agonist NECA also elicited a concentration-dependent increase in luminescence (Fig. 1b). Whilst the magnitude of raw luminescence observed was dependent on biosensor expression level and GloSensor™ substrate concentration in a given experimental plate, the luminescent output afforded by 10 μM NECA was consistently greater (P < 0.05, unpaired *t*-test) than that of 10 μM forskolin and both were considerably greater than that observed with vehicle (HBSS) alone (Fig. 1a).

**Fig. 3:**
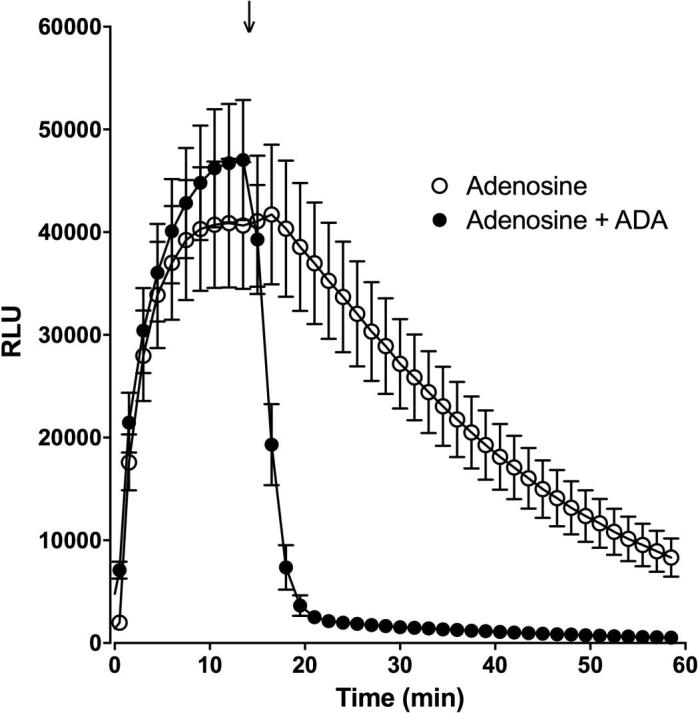
The influence of adenosine deaminase on the kinetic profile of adenosine mediated cAMP production. Glosensor luminescence time course following stimulation by 1 mM adenosine and subsequent addition of HBSS (open symbols) or 2 U.ml^−1^ adenosine deaminase (ADA; closed symbols) at the peak luminescence obtained (arrow). Data represent mean ± S.E.M. of 6 separate experiments each performed in triplicate, RLU; raw luminescence units.

It was evident that the rate of luminescent decay at the two highest NECA concentrations tested (10–30 µM) was markedly greater than that observed with a lower concentration of NECA (3 μM) giving an equivalent sized response (Fig. 2b). This was not seen with forskolin (Fig. 2a). The most likely explanation for this phenomenon is that receptor desensitization is occurring at the highest concentrations of NECA employed in this study. To investigate the rate of decline of the GloSensor™ response upon termination of A_2B_AR stimulation, as would occur when the agonist is removed or the receptor desensitized, we investigated the effect of addition of adenosine deaminase (ADA), an enzyme that metabolises adenosine to inosine, on an established adenosine response (Fig. 3). The addition of 2 U ml^−1^ ADA at the peak response to adenosine rapidly reduced the luminescence output to basal levels within a few minutes (Fig. 3). In contrast, addition of HBSS in place of ADA resulted in a slow decline (Fig. 3) similar to that seen with forskolin or 3 μM NECA alone (Fig. 2a-b).

### 3.2 Characterisation of A_2B_AR and A_2A_AR responses in the HEK293G cell line

**Fig. 4:**
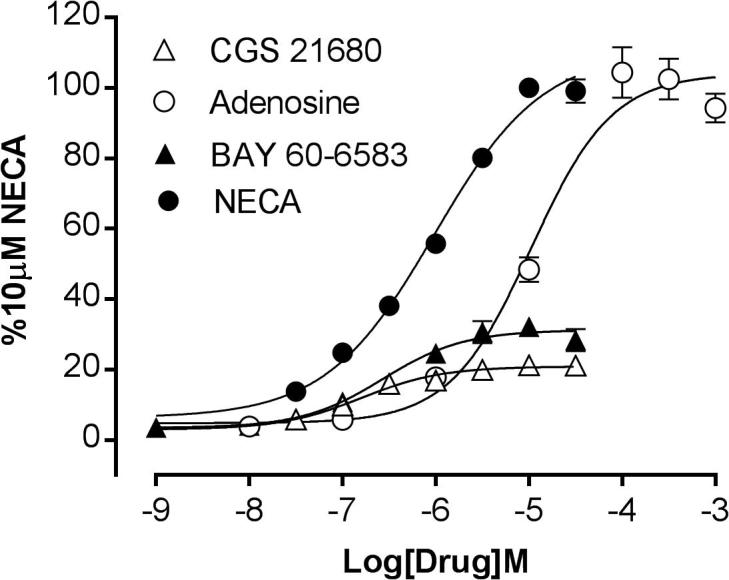
Concentration response curves for agonist-stimulated cAMP Glosensor luminescence responses in HEK 293 cells giving pEC50s of 6.05 ± 0.08 (n = 21, NECA), 4.69 ± 0.08 (n = 13, adenosine), 6.19 ± 0.02 (n = 3, BAY 60-6583) and 6.45 ± 0.06 (n = 13, CGS 21680). Data represent mean ± S.E.M of the peak luminescence response in individual experiments carried out in triplicate. Data are expressed as a percentage of the peak luminescence response obtained with 10 µM NECA measured in the same experiment.

Concentration-response curves for agonist-stimulated GloSensor™ responses are shown in Fig. 4, with the selective A_2B_ agonist BAY 60-6583 behaving a partial agonist relative to that of the endogenous agonist adenosine and its analogue NECA. Most notably the A_2A_AR-selective agonist CGS 21680 was able to stimulate a small but significant response within the HEK293G cell line (18.6 ± 4.5% that obtained with NECA; n = 12; P < 0.01; paired *t* test relative to basal), suggesting that the HEK293G cell line contained a mixed population of A_2A_AR and A_2B_AR. Interestingly, BAY 60-6583 gave a similar time course (providing evidence of desensitization at higher agonist concentrations) to that observed with NECA (Fig. 2c) whilst CGS 21680 did not (Fig. 2d). In addition the concentration-response curve for NECA had a Hill slope significantly less than unity (0.75; p < .001; Partial F test) which would be consistent with the presence of more than one component, similar to that seen previously in HMC-1 cells, which also expresses both A_2A_AR and A_2B_AR subtypes [32].

**Fig. 5:**
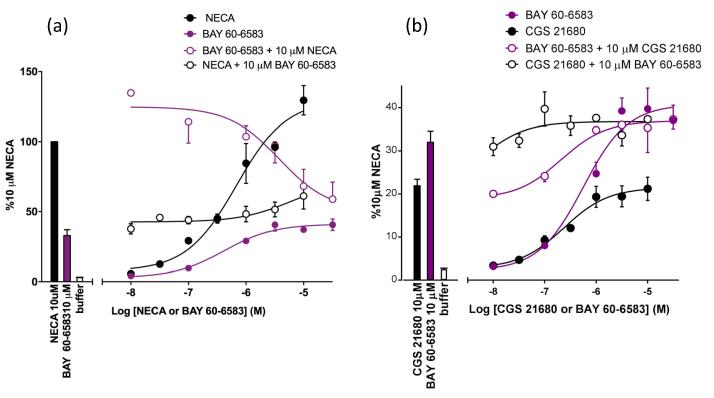
Concentration response curves following (a) BAY 60-6583 or NECA addition and (b) BAY 60-6583 or CGS 21680 addition in the presence (open symbol) or absence (closed symbol) of 10 µM of the alternate agonist. Data represent mean ± S.E.M of the peak luminescence response in individual experiments carried out in triplicate. Data are expressed as a percentage of the peak luminescence response to 10 μM NECA obtained in three separate experiments. In each individual experiment triplicate determinations were made.

The partial agonist nature of the response to the A_2B_-selective agonist BAY 60-6583 at the A_2B_AR was confirmed in combination experiments with NECA (Fig. 5a). Thus, increasing concentrations of BAY 60-6583 were able to antagonise the response to a fixed concentration of NECA (10 µM) whilst a fixed concentration of BAY 60-6583 (10 µM) was able to shift the concentration-response curve to NECA to higher agonist concentrations (Fig. 5a). In marked contrast, the responses to the A_2B_-selective agonist BAY 60-6583 and the A_2A_ selective agonist CGS 21680 were simply additive at low concentrations of each agonist, but reached a maximum that appeared to be entirely determined by the maximum response to BAY 60-6583 (Fig. 5b). These data suggest that the overall response to a combination of A_2A_ and A_2B_ receptor stimulation is largely determined by the maximum A_2B_ signal in this cell line.

**Fig. 6:**
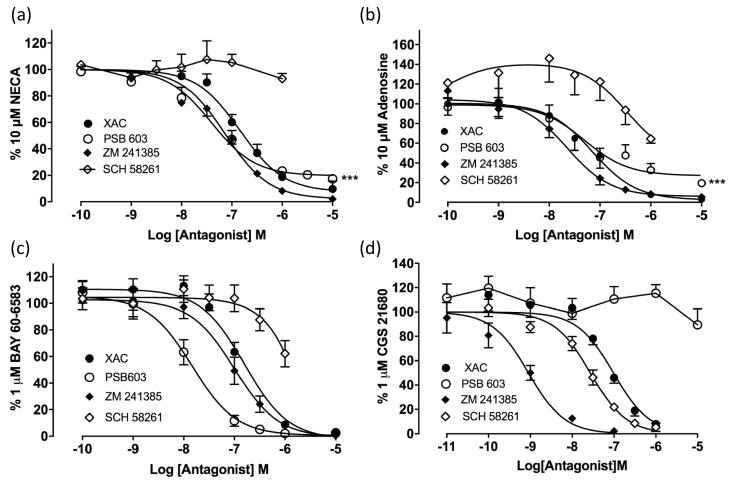
Antagonism of agonist-stimulated cAMP Glosensor responses by increasing concentrations of adenosine receptor antagonists. Competition curves for (a) NECA (10 µM), (b) adenosine (10 μM), (c) BAY 60-6583 (1 μM) or (d) CGS 21680 (1 μM) stimulated cAMP responses following 30 min pre-incubation with XAC, PSB 603, ZM 241385 or SCH 58261. Data represent mean ± S.E.M of the peak luminescence response in individual experiments carried out in triplicate. Data are expressed as% of the peak luminescence response to each agonist obtained in the absence of antagonist for (a) seven (PSB), six (XAC), five (ZM 241385) or four (SCH 58261) separate experiments performed in triplicate. The residual response following 10 µM PSB 603 pre-incubation is significantly different to that seen with following stimulation with vehicle alone (1 way ANOVA, ^***^P < .001); (b) of four (XAC), five (PSB 603, ZM 241385) or six (SCH 58261) separate experiments performed in triplicate. The residual luminescence response following 10 µM PSB 603 pre-incubation is significantly different to that seen with following stimulation with vehicle alone (1 way ANOVA, ^***^P < .001); (c) five (XAC, ZM 241385, SCH 58261) or seven (PSB 603) separate experiments performed in triplicate; (d) five (XAC), six (PSB 603), seven (SCH 58261) or eight (ZM 241385) separate experiments performed in triplicate.

**Fig. 7:**
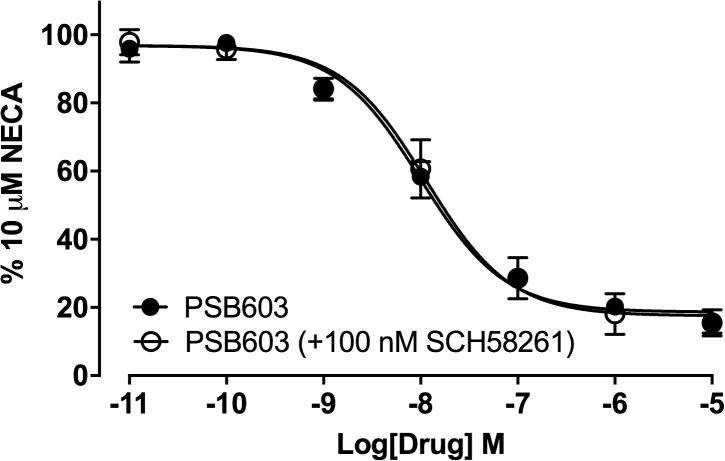
The influence of SCH 58261 on the inhibition of NECA-stimulated cAMP production by PSB 603. Inhibition of the peak NECA (10 μM) stimulated responses following 30 min pre-incubation with the A2b selective antagonist PSB 603 (n = 4) in the presence or absence of the A2a selective antagonist SCH 58261 (100 nM). Data represent mean ± S.E.M of the peak luminescence response in individual experiments carried out in triplicate. Data are expressed as a percentage of the peak luminescence response to10 μM NECA obtained in the absence of antagonist in each individual experiment.

To investigate this further we examined the effect of subtype-selective antagonists on the responses to 10 µM NECA, 10 μM adenosine, 1 μM BAY 60-6583 or 1 μM CGS 21680 (Fig. 6). Pre-incubation with the non-selective adenosine receptor antagonist XAC inhibited the response to 10 µM NECA in a concentration-dependent manner, completely abolishing the response at 10 µM XAC (Fig. 6a). Similarly, the highly selective A_2B_AR antagonist PSB 603 produced a concentration-dependent inhibition of the NECA response, however the maximal inhibition achieved was only 81.7 ± 2.4% (n = 7, 1-way ANOVA P < .001 when compared to the response following stimulation with vehicle alone) of the maximal response to NECA (Fig. 6a). It is unlikely that this residual response following pre-incubation with the highest PSB 603 concentration is due to an A_2A_AR population since the A_2A_AR antagonist ZM 241385 also produced a concentration dependent inhibition of the response to NECA with complete inhibition at 10 µM (Fig. 6a). However the equilibrium dissociation constant obtained following ZM 241385 pre-incubation was entirely consistent with an A_2B_AR-mediated response (Table 1; [24], [33]). Consistent with this suggestion, the more selective A_2A_AR antagonist SCH 58261 [34] failed to inhibit the response to NECA at all concentrations studied (Fig. 6a). Inclusion of 100 nM SCH 58261 in the competition curve obtained with PSB 603 did not produce a greater maximal inhibition of the response to NECA (Fig. 7).

Antagonism of the response to adenosine and BAY 60-6583 produced profiles that resembled that of NECA (Fig. 6b-c). Similar to the data obtained with NECA, 10 µM PSB 603 produced only 80.5 ± 3.0% inhibition of the response to 10 µM adenosine (Fig. 6b, 1-way ANOVA P < .001 when compared to the response following stimulation with vehicle alone). SCH 58261 appeared to enhance the production of cAMP at lower concentrations whilst inhibiting approximately 35% of the adenosine response at the highest concentration tested, although neither effect was statistically significant. SCH 58261 was a weak antagonist of the response to BAY 60-6583. The response to this agonist was potently antagonised by PSB 603 and was able to completely abolish the response to BAY 60-6583 (Fig. 6c). Typical A_2A_AR pharmacology was observed for responses to the selective A_2A_AR agonist, CGS 21680 (Fig. 6d). XAC, ZM 241385 and SCH 58261 conferred complete concentration-dependent antagonism of the CGS 21680 response. The highly selective A_2B_AR antagonist, PSB 603, was unable to inhibit the CGS 21680 response at any of the concentrations tested. Apparent equilibrium dissociation constants for the different antagonists from this analysis are shown in Table 1.

### 3.3 Mechanism of A_2B_AR antagonism by PSB 603

**Fig. 8:**
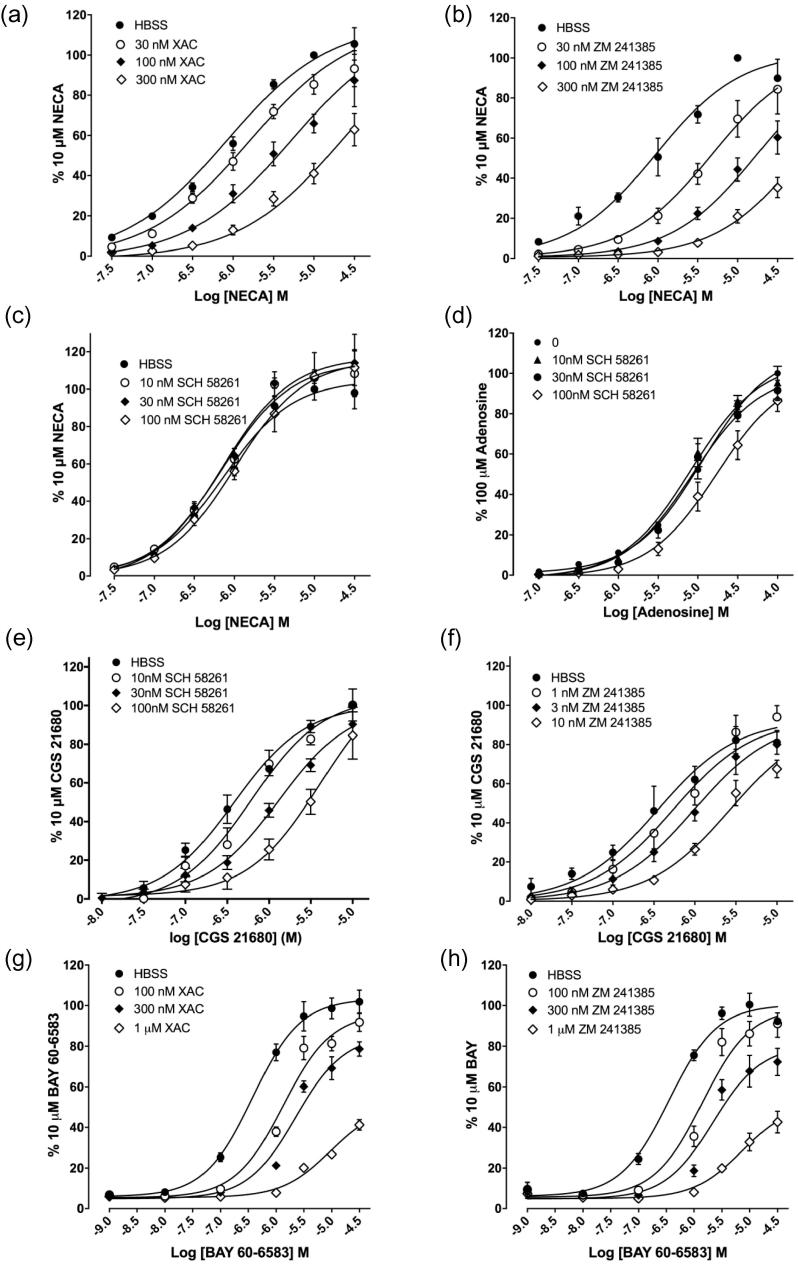
Influence of adenosine receptor antagonists on concentration-response curves to NECA, Adenosine, CGS 21680 and BAY 60-6583. NECA (a-c), Adenosine (d), CGS 21680 (e-f) and BAY 60-6583 (g-h) concentration response curves were obtained in the presence and absence of increasing concentrations of (a,g) XAC, (b,f,h) ZM 241385 or (c-e) SCH 58261. Values are mean ± S.E.M. of (a,f-h) five, (b,d) four or (c,e) three separate experiments carried out in triplicate. Data represent peak luminescence response and are expressed as a percentage of the peak luminescence response to 10 μM NECA, 100 μM Adenosine, 10 μM CGS 21680 or 10 μM BAY 60-6583 obtained in the absence of antagonist in each individual experiment.

In order to further characterise the mechanism of action of these antagonists at the adenosine A_2_ receptor population within HEK293G cells, concentration-response curves were obtained for NECA, adenosine, BAY 60-6583 and CGS 21680 following pre-incubation with increasing concentrations of different antagonists. Pre-incubation with increasing concentrations of both XAC and ZM 241385 elicited parallel rightwards shifts of the concentration-response curves to NECA (Fig. 8a, b) consistent with a competitive interaction between agonist and antagonist at the A_2B_AR. Analysis of these shifts yielding pA2 (−log K_B_) values of 7.50 ± 0.09 (n = 4) and 8.02 ± 0.10 (n = 3) for XAC and ZM 241385 respectively. These are similar to the values obtained in Table 1. In contrast, the A_2A_AR -selective antagonist SCH 58261 did not shift the NECA concentration response curve to higher agonist concentrations, and only shifted the adenosine response curve at the highest concentration (100 nM) tested (Fig. 8c-d). However, both SCH 58261 and ZM 241385 competitively antagonised the responses to the A_2A_-selective agonist CGS 21680 (Fig. 8e,f) yielding pA2 (-log K_B_) values of 7.79 ± 0.15 (n = 3) and 8.64 ± 0.12 (n = 5) for SCH 58261 and ZM 241385 respectively. Increasing concentrations of XAC and ZM 241385 (Fig. 8g-h) both gave sequential rightwards shifts of the concentration response curve for BAY 60-6583 at lower antagonist concentrations but significantly depressed the E_max_ at the highest antagonist concentrations used (Table 3; 1 µM, p < .0001 Fig. 8g, p < 0.001Fig. 8h).

**Table 3:** BAY 60-6583 pEC_50_ and E_max_ values obtained in the presence of increasing concentrations of XAC or ZM 241385.

	XAC		ZM 241385	
Antagonist (nM)	pEC_50_	E_max_	pEC_50_	E_max_
0	6.42 ± 0.04	103.50 ± 5.27	6.46 ± 0.08	100.80 ± 4.61
100	5.88 ± 0.06^**^	96.56 ± 4.80	5.85 ± 0.06^***^	99.03 ± 7.14
300	5.65 ± 0.04^***^	85.63 ± 5.04	5.67 ± 0.06^****^	80.84 ± 8.36
1000	5.03 ± 0.18^****^	55.89 ± 7.86^****^	5.14 ± 0.12^****^	54.08 ± 8.41^***^

pEC_50_ and E_max_ values obtained for BAY 60-6583 obtained in the presence of increasing concentrations of XAC or ZM 241385. E_MAX_ values are expressed as a percentage of the response obtained with 10 μM BAY 60-6583. Significant differences to that seen in the absence of antagonist with each agonist are indicated (^**^p < .01, ^***^p < .001,^****^p < .0001 1-way ANOVA). Data are expressed as mean ± S.E.M. of 5 separate experiments.

**Fig. 9:**
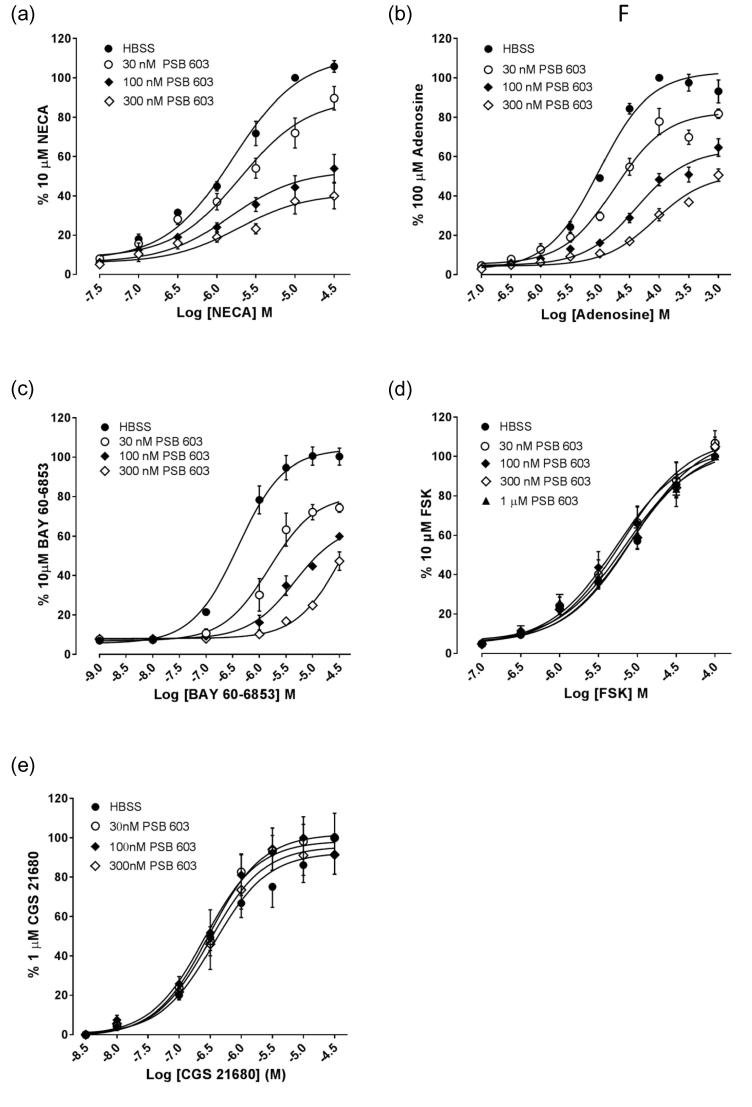
Influence of PSB 603 on concentration-response curves to adenosine receptor agonists and forskolin**.** Influence of increasing concentrations of PSB 603 on the agonist concentration-response curves for (a) NECA, (b) Adenosine (c) BAY 60-6583, (d) forskolin (FSK) and (e) CGS 21680. Values represent mean ± S.E.M. obtained in (a) four, (b,e) seven or (c,d) six separate experiments carried out in triplicate. Data represent the peak luminescence response and are expressed as a percentage of the peak luminescence response obtained with 10 μM NECA, 10 μM adenosine, 1 μM BAY 60-6583, 10 μM FSK or 1 μM CGS 21680 in the absence of antagonist in each individual experiment.

Increasing concentrations of the A_2B_AR selective antagonist PSB 603 significantly depressed the maximum response to NECA (p < .01; Table 2; one-way ANOVA) but did not significantly affect the EC_50_ (Fig. 9a, Table 2). PBS 603 also appeared to depress the maximum response to adenosine (p < .01; Table 2), however this was accompanied with a small significant shift of the EC_50_ value to higher against concentrations (Fig. 9b, Table 2). PSB 603 produced a larger shift in the EC_50_ for BAY 60 6583 (Fig. 9c) although the effect on the smaller maximum response of BAY 60 6583 was not well defined (Table 2). PSB 603 pre-incubation, however, did not affect the response to forskolin (Fig. 9d) or CGS 21680 (Fig. 9e).

**Fig. 10:**
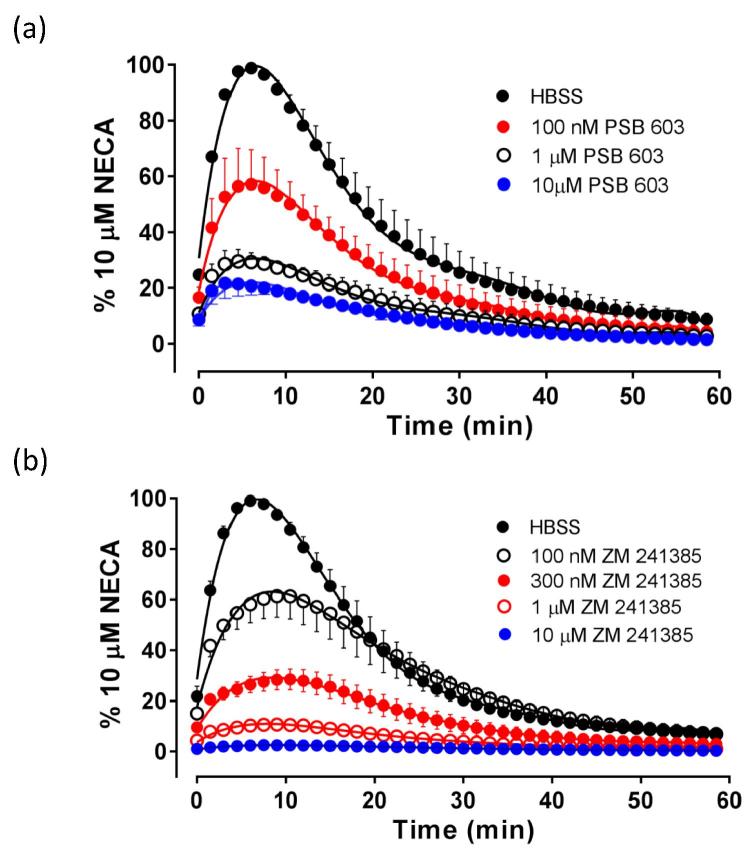
Kinetic profile of NECA-stimulated cAMP GloSensor luminescence obtained in the presence of increasing concentrations of (a) PSB 603 or (b) ZM241385. Data (mean ± S.E.M.) are expressed as a percentage of the peak luminescence response to 10 μM NECA obtained in the absence of antagonist in six (PSB 603) or five (ZM 241385) separate experiments. In each experiment triplicate determinations were made.

The non-competitive antagonism of the NECA- and adenosine-mediated stimulation of cAMP production by PSB 603 suggests a negative allosteric mode of action at A_2B_ARs. This is consistent with the incomplete attenuation of the response to NECA and adenosine shown in Fig. 6a and b, indicative of a saturable allosteric effect. To further investigate this phenomenon, we have also looked in detail at the kinetics of the response to NECA obtained in the presence of different concentrations of PSB 603 (Fig. 10). In comparison to the kinetics of the antagonism obtained with ZM 241385, there are two characteristics of the kinetics obtained with NECA in the presence of PSB 603 which are notable: (1) the antagonism by PSB 603 of the peak response to NECA reaches a limiting level leaving a residual response (*circa* 20%) (Fig. 10a) that is not observed with ZM 241385 (Fig. 10b) and (2) the time to the peak NECA response decreases in the presence of increasing concentrations of PSB 603 (Fig. 10a).

## 4 Discussion

In the present study we have used a real-time kinetic assay of changes in cAMP-stimulated PKA activation (GloSensor™; [25], [26]) to investigate the molecular pharmacology of the A_2B_AR -selective antagonist PSB 603 [6], [16] in HEK 293 cells, endogenously expressing the human A_2B_AR [21]. The GloSensor™ biosensor provided a robust kinetic profile of the responses to forskolin and NECA that were concentration dependent and characterised by a peak in luminescence occurring between 5 and 20 min.

BAY 60-6583 acted as a partial agonist of the A_2B_AR cAMP response in these cells. This was evident from both the lower maximum response obtained relative to NECA and the ability of high concentrations of BAY 60-6583 to antagonise the response to a high concentration of NECA (Fig. 5a). Hinz et al., have also observed partial agonism with BAY 60-6583 in both endogenous and transfected lines [35], although Thim et al. [6] reported a full agonist response which would be expected at the higher receptor expression levels achieved with retroviral transfection approaches.

A notable feature of the response to high concentrations of NECA and BAY 60-6583 was that the decay in GloSensor™ luminescence was more rapid than that seen with lower agonist concentrations or indeed with all concentrations of forskolin. This is most likely a consequence of receptor desensitization at the high concentrations of NECA or BAY 60-6583 employed and suggests that the GloSensor™ system is accurately reporting the dynamic state of cAMP levels within the cells, and hence the extent of receptor activation. To examine receptor activation more directly we were able to use adenosine as the agonist and show that the luminescence signal rapidly declines to basal levels when the adenosine metabolising enzyme adenosine deaminase is added at the peak of the agonist response. Without the continued stimulus on the active receptor, residual cAMP is rapidly exported from the cell and/or hydrolysed by phosphodiesterases. It follows therefore that the increased rate of luminescent decay observed with the higher NECA or BAY 60-6583 concentrations could result from a reduced pool of active receptor which would be apparent due to desensitization of the receptor. This A_2B_AR rapid agonist-driven desensitization has been noted previously and has been shown to be mediated by GRK2 that leads to β-arrestin-2 dependent receptor internalisation and subsequent re-sensitization or degradation [36], [37], [38], [39].

Consistent with previous reports [21], a pharmacological analysis of the GloSensor™ responses to NECA and adenosine indicated that they were largely mediated by A_2B_ARs. The responses were antagonised by the A_2B_AR selective antagonist PSB 603. The selective A_2A_AR antagonist ZM 241385 only antagonised responses to NECA and adenosine at higher concentrations (pK_i_ = 8.1 and 7.9 respectively; Table 1) than those required to selectively inhibit the A_2A_AR (pKi > 9.0; [24], [33]). Furthermore, the more selective A_2A_AR antagonist SCH 58261 [34] failed to inhibit the response to NECA at all concentrations studied in keeping with its known affinity for the A_2B_AR (pKi 6.0; [24]). A small inhibition of the responses to BAY 60-6583 and adenosine by SCH 58261 was only observed at the highest concentration of this A_2A_AR-antagonist used. This is likely to be due to the fact that SCH 58261 will start to inhibit the A_2B_ receptor at concentrations in the micromolar range. The A_2A_AR -selective agonist CGS 21680, however, was able to stimulate a small response in these cells (18.6% of that achieved with NECA) and this had an antagonist profile (Fig. 6d) consistent with an A_2A_AR -mediated response. This response to CGS 21680 was antagonised competitively by ZM 241385 and SCH 58261 with pKi values of 9.4 and 8.2 respectively (Fig. 8e) but was not affected by PSB 603 at concentrations up to 10 μM. Taken together, these data are consistent with the presence of both A_2A_ARs and A_2B_ARs in HEK 293G cells regulating the formation of cAMP.

It was notable that both XAC and ZM241385 decreased the E_MAX_ for BAY 80-6583 at the highest concentrations used. This is likely to be a result of a combination of: (a) the partial agonist nature of BAY 60-6583; (b) the transient nature of the Glosensor cAMP response produced by BAY 60-6583 and (c) the long residence time on the receptor of the two antagonists at high concentrations leading to hemi-equilibrium conditions. i.e. At the highest concentration of XAC and ZM241385 used, it is likely that the antagonist dissociates too slowly to allow the increasing agonist concentrations to reach equilibrium and overcome the antagonism before the agonist response wanes.

The presence of a 20% contribution from A_2A_AR to the cAMP responses to NECA and adenosine (predicted by the data obtained with CGS 21680) in this cell line suggests that only an 80% inhibition of these latter two agonists would be expected with the highly selective A_2B_AR antagonist PSB 603. This was what was observed (Fig. 6). However, inclusion of 100 nM SCH 58261 along with PSB 603 (Fig. 7) gave no indication of an A_2A_AR component in the response to NECA. This was also the case when concentration-response curves to NECA were analysed in the presence of increasing concentrations of SCH 58261 (Fig. 8c). These data suggest that cAMP responses to NECA and adenosine are only mediated via the A_2B_AR in this endogenously expressing cell line. The most likely explanation for the failure to observe a significant A_2A_AR component in the responses to NECA and adenosine is that a rapid A_2A_AR heterologous desensitization occurs [40] as a result of the activation of A_2B_AR.

The ability of A_2B_AR activation to over-ride any concomitant activation of A_2A_AR is also suggested by the data obtained with BAY 60-6583 and CGS 21680 in combination (Fig. 5). In Fig. 5a a classical demonstration of the partial agonist effect of BAY 60-6583 was demonstrated. However, in Fig. 5b where the interaction between BAY 60-6583 and CGS 21680 is investigated, the responses to the two agonists were simply additive at low concentrations of each agonist, but reached a maximum that was entirely determined by the maximum response to BAY 60-6583. These data support the contention that the overall response to a combination of A_2A_ and A_2B_ receptor stimulation is largely determined by the larger A_2B_ signal in this cell line.

An alternative explanation for the limited 80% inhibition of the cAMP responses to NECA and adenosine observed with PSB 603 (but not with XAC or ZM 241385) is that it is acting allosterically and the negative cooperative effect reaches a saturable effect [17], [18], [19], [20]. In keeping with this hypothesis, the effect of increasing concentrations of PSB 603 on the concentration responses curves to NECA (Fig. 9a) and adenosine (Fig. 9b) were not consistent with competitive antagonism. In the case of NECA, the maximal response was significantly decreased by increasing concentrations of PSB 603 without significant effect on the pEC_50_ values (Table 2). The decrease in maximal response obtained with PSB 603 was not a consequence of off-target effects since concentration-response curves to both forskolin and CGS 21680 were completely unaffected at concentration of PSB 603 up to 300 nM. Furthermore, the small shift in pEC_50_ that accompanied the marked reduction in E_MAX_ observed in response to adenosine is consistent with the probe dependence (i.e. which ligand is occupying the orthosteric site) of an allosteric mechanism of action [17], [18], [19], [20].

As previously reported [35], BAY 60-6583 acted as a partial A_2B_AR agonist in the present studies. Interestingly, PSB 603 was able to completely attenuate the Glosensor responses to this lower efficacy agonist. However, as with the more efficacious agonists NECA and adenosine, the antagonism produced by PSB 603 of the BAY 60-6583 responses was not completely compatible with a simple competitive interaction. These observations would be compatible with the probe dependence referred to above [17], [18], [19], [20].

Analysis of the kinetic profiles of the responses to NECA in the presence and absence of PSB 603 or ZM 241385 also suggested a different mechanism of action for these two antagonists. In comparison to the kinetics observed with ZM 241385, the antagonism by PSB 603 reached a limiting level (evidence of saturation) leaving a residual response (*circa* 20%) to NECA that was not observed with ZM 241385. Furthermore, the peak response to NECA was obtained at shorter incubation times in the presence of increasing concentrations of PSB 203. This was not observed with ZM 241385 where the peak responses to NECA in the presence of 100 or 300 nM ZM 241385 were obtained at later time points, as would be expected for a competitive antagonist. Taken together, these data suggest that the mechanism of action of PSB 603 at the A_2B_AR is due to a negatively cooperative effect on the binding affinity and/or efficacy of agonists acting via the orthosteric site. Previous works with ^3^H-PSB-603 [6], [16] have provided some evidence that PSB 603 may bind to a different set of amino acids to those used by the endogenous ligand adenosine, and this would be consistent with an allosteric mechanism of action.

In conclusion, the data presented here suggest that PSB 603 acts as a highly selective negative allosteric modulator of A_2B_AR -mediated increases in cAMP accumulation in HEK 293 cells endogenously expressing the human A_2B_AR. Interestingly, other positive and negative allosteric modulators of this receptor have recently been reported [41]. Allosteric modulation of the human A_2B_AR therefore represents a novel route to the development of therapeutic agents to treat conditions such as inflammation and ischemia where adenosine levels can be quite high (and could reduce the effectiveness of competitive receptor antagonists).
